# On the Role of Testosterone in Anxiety-Like Behavior Across Life in Experimental Rodents

**DOI:** 10.3389/fendo.2018.00441

**Published:** 2018-08-06

**Authors:** Emese Domonkos, Július Hodosy, Daniela Ostatníková, Peter Celec

**Affiliations:** ^1^Faculty of Medicine, Institute of Molecular Biomedicine, Comenius University, Bratislava, Slovakia; ^2^Faculty of Medicine, Institute of Physiology, Comenius University, Bratislava, Slovakia; ^3^Faculty of Medicine, Institute of Pathophysiology, Comenius University, Bratislava, Slovakia; ^4^Department of Molecular Biology, Faculty of Natural Sciences, Comenius University, Bratislava, Slovakia

**Keywords:** anxiety-like behavior, perinatal, adolescence, puberty, aging

## Abstract

Testosterone affects brain functions and might explain some of the observed behavioral sex differences. Animal models may help in elucidating the possible involvement of sex hormones in these sex differences. The effects of testosterone have been intensively investigated, especially in anxiety models. Numerous experiments have brought inconsistent results with either anxiolytic or anxiogenic effects. Besides methodological variations, contradictory findings might be explained by the divergent metabolism of testosterone and its recognition by neurons during prenatal and postnatal development. Gonadectomy and subsequent supplementation have been used to study the role of sex hormones. However, the variable duration of hypogonadism might affect the outcomes and the effect of long-term androgen deficiency is understudied. Testosterone can be metabolized to dihydrotestosterone strengthening the androgen signaling, but also to estradiol converting the androgen to estrogen activity. Moreover, some metabolites of testosterone can modulate γ-aminobutyric acid and serotonergic neurotransmission. Here we review the currently available experimental data in experimental rodents on the effects of testosterone on anxiety during development. Based on the experimental results, females are generally less anxious than males from puberty to middle-age. The anxiety-like behavior of females and males is likely influenced by early organizational effects, but might be modified by activational effects of testosterone and its metabolites. The effects of sex hormones leading to anxiogenesis or anxiolysis depend on factors affecting hormonal status including age. The biological and several technical issues make the study of effects of testosterone on anxiety very complex and should be taken into account when interpreting experimental results.

## Introduction

Besides depression, anxiety disorders, including generalized anxiety disorder, panic disorder, phobias, social anxiety disorder, separation anxiety disorder, obsessive-compulsive disorder and post-traumatic stress disorder, are the most common of all mental disorders (1). According to a survey performed in 2010, 14% of the population in Europe suffers from an anxiety disorder each year (2). The prevalence rate of anxiety disorders is influenced by various factors, including gender (3). It is known that sex hormones affect brain morphology as well as brain functions. Based on the sex differences in prevalence of anxiety disorders in human, the role of sex hormones has been intensively investigated. A large body of animal experiments has brought contradictory results showing either anxiolytic or anxiogenic effects of testosterone (4, 5). These inconsistent findings might be due to differences in organizational and activational actions of testosterone affecting the neuronal circuits during prenatal and postnatal development. Therefore, in the present paper, the currently available experimental data on the sex differences in anxiety-like behavior and the potential role of testosterone affecting these differences across different period of life are reviewed.

## Sex differences in anxiety-like behavior in rodent models

Anxiety is interconnected with behavioral and brain functions such as fear response. In clinical practice, anxiety is considered to be a consequence of overestimated response to threat in uncertain situations associated with prolonged hyper-vigilance and hyper-arousal (6). In neuropsychiatric studies, rodents are the most commonly used animal models (7). While in humans, anxiety might be assessed by self-report questionnaire, in rodents anxiety is evaluated based on temporary behavioral responses to various threatening stimuli. Therefore the translational value of such evaluation is disputable. On the other hand, animal experiments are crucial to elucidate causality behind observed associations. Although mice and rats may differ in some behavioral traits, the behavioral tests assessing anxiety in rodents are based on their common innate avoidance of certain conditions, such as bright illuminations (8) or open space (9–11). Therefore the most of the anxiety tests are suitable for both, mice and rats (7). In anxiety assessment it should be also considered that anxiety is associated with other behavioral phenotypes, such as aggression and violence (12–19). These exhibit sex differences and positive correlations with testosterone concentration (20, 21). Furthermore, anxiety-related behavior assessed in experimental rodents is often related to their cognitive performance, which might be affected by sex hormones (22, 23) and dependent on androgen signaling (24).

It should be noted that some behavioral traits, including anxiety, might be influenced by estrous cycle in females. It has been shown that female rat in proestrus display more explorative behavior in a novel environment and less freezing behavior following shock in defensive burying test, they spent more time in the open arm of the elevated plus maze and interact longer with an unfamiliar social partner in comparison to males but also to females during other phases of the estrous cycle (25). Although, to cover whole estrous cycle, females in the experiments are often selected for each stages and a heterogeneous group is used, but it leads to high inter-individual variability (26–28). Therefore, the assessment of sex differences in anxiety behavior is problematic.

**Figure 1:**
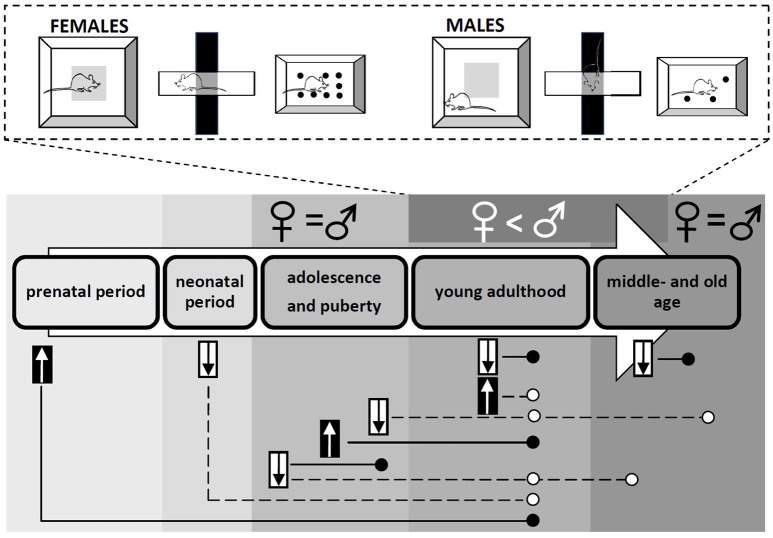
Sex differences in anxiety-like behavior assessed using the open field, elevated plus maze and marble-burying tests. The consequences of testosterone exposure and deprivation in males are shown across the life. 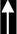 - testosterone exposure; 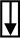 - testosterone deprivation; ° - anxiolytic effect; • - anxiogenic effect.

Women are twice more likely to experience anxiety as men (26). However, it seems that sex differences in the behavioral stress responses mostly point to the opposite direction. Despite of the evidence suggesting the effect of hormonal fluctuation, the behavior of the females is often generalized without considering the estrous cycle, especially in the earlier experiments investigating sex differences. Such results show that adult female rats move in the open field more than males (29), while male rats display more freezing behavior and defecation, longer-lasting grooming reaction, less rearing as well as less time spent in the center zone of the arena (28). Females, mice as well as rats, make more entries into the open arms and spent greater time in open arms of the elevated plus maze, interact more with a same-sex social partner in a novel environment and bury fewer marbles than males (27, 28, 30, 31). On the contrary, in the Vogel conflict test, male rats seems to be less anxious than females indicated by more licking of the drinking spout during the punish period (30). In time spent in the bright chamber of the light-dark box, contradictory results have been found. In some experiments, females show higher anxiety than males (32), in other experiments, the opposite sex difference was proved (33), while the lack of sex differences is published rarely (27). It should be noted that sex differences in experimental animals in anxiety-like behavior might depend on many factors, such as strain (34) or breeding condition (32), and other sources of inter-individual variability are likely not identified yet (35). The sex differences in frequently used behavioral tests for anxiety changing with age, as well as the short- and long-term consequences of testosterone deprivation or exposure in males in different periods of life are illustrated in Figure 1.

## The role of testosterone in anxiety-like behavior of rodents across different life periods

The sexually dimorphic behaviors related to anxiety can be influenced by both, organizational and activational effects of gonadal hormones. Organization of the neuronal circuits by testosterone and/or its metabolites predetermines the behavioral responses to the activational effects of sex hormones later in life. Besides the sensitive period of early—prenatal and perinatal development (15, 27), increasing amount of evidence supports the hypothesis that pubertal hormones (re)organize the brain and determine a variety of adult behaviors during adolescence (36, 37) or outside of the classic critical periods of neurodevelopment (38). An overview of the results of some experiments investigating sex differences and the role of testosterone in anxiety-like behavior, with particular regard to the age of animals with hormone manipulation and analysis of the outcomes, is provided in Table 1.

**Table 1:** Overview of some experimental results on the effect of testosterone on anxiety-like behavior in regard to age at hormone manipulation.

**References**	**Species/Gender**	**Age at intervention**	**Intervention**	**Age at anxiety examination**	**Anxiety examination**	**Outcome**
**PRE- UND PERINATAL PERIOD**						
(39)	Rat (Wistar) Females and males	Prenatal period (GD 15–19)	Prenatal androgen exposure (PNA): pregnant dams administered with testosterone (0.5 mg/kg/d)	Young adulthood (PND 53-59)	Elevated plus maze	PNA offspring, particularly females, showed anxiety-like behavior
(28)	Rat (Wistar) Females and males	Prenatal period (GD 15–20)	Prenatal inhibition of testosterone aromatization: pregnant dams administered with 1,4,6-androstatriene-3.17-dione (ATD; aromatase inhibitor, 5 mg/rat)	Adolescence and young adulthood (1 and 3 months)	Open field, elevated cross maze	Gender differences: females were more active, less anxious and emotional than males at 3 months prenatal ATD resulted in increases in anxiety and emotionality, which in males depended on the age of examination
(27)	Mice (129:C57BL/6J) Females and males	Perinatal period (day of birth)	Testosterone injected in females (100 μg/pup)	Young adulthood (PND 67–78)	Marble-burying test, light-dark box	Gender differences: females were less anxious than males in marble-burying single injection of testosterone masculinized female anxiety-related behavior in marble-burying test
(40)	Rat (Long Evans) Males	Perinatal period (day of birth)	Gonadectomy	Young adulthood (ca. PND 120)	Open field, novel object exposure, light-dark box, elevated plus maze	Neonatal gonadectomy had anxiolytic effect in adult rats
**PERIPUBERTAL AGE**						
(26)	Mice (C57BL/6N) Females and males	Pre-pubertal age (PND 24-25)	Gonadectomy	Before puberty (PND 24) and late puberty (PND 40-47)	Elevated plus maze, open field, marble-burying test	Gender differences: in late adolescence males were less anxious than females, these gender differences were not found prior puberty or in young adulthood males as well as females showed an increase in anxiety-like behavior in marble-burying test from pre-pubertal to late-pubertal age; pre-pubertal gonadectomy had anxiogenic effect in males, but anxiolytic effect in females at late-pubertal age
(27)	Mice (129:C57BL/6J) Females and males	Pre-pubertal age (PND 28)	Ovariectomy + testosterone capsules implanted in females (3 mm/mouse)	Young adulthood (PND 67-78)	Marble-burying test, light-dark box	Testosterone treatment masculinized female anxiety-related behavior in marble-burying test
(41)	Rats (Lewis) Females and males	Pre-pubertal age (PND 29-31)	Gonadectomy in males	Middle-age (from 12 months)	Open field, light-dark box, elevated plus maze, PhenoTyper cage	Gender differences: females were less anxious than males in most of the conducted tests long-term androgen deficiency decreased sex differences (anxiolytic effect in males);
(33)	Rat (Lister hooded) Females and males	pre-pubertal age (PND 33-34) and post-pubertal age (PND 58-59)	Gonadectomy in males	Young adulthood (PND 101–110)	Elevated plus maze, light-dark box, open field	Gender differences: at PND 95–96, intact males were more anxious than intact females in light-dark box pre-pubertally castrated males displayed less anxious behavior than post-pubertally castrated males
(42)	Rats (Wistar) Females and males	Post-pubertal age (PND 47)	Gonadectomy in males	Old-age (30 months)	Open field, light-dark box, elevated plus maze, PhenoTyper cage	No gender differences were found in anxiety-related behavior at old age except the light-dark box (anxiolytic effects) gonadectomy had no effect on anxiety in males
**MIDDLE- AND OLD AGE**						
(22)	Rats (Fisher) Males	Young adulthood (4 months), middle-age (13 months) and old-age (24 months)	Gonadectomy + testosterone capsules implanted (10 mm/rat) in gonadally intact male rats: testosterone (1 mg/kg) 3α-diol (1 mg/kg)	Young adulthood (4 months), middle-age (13 months) and old-age (24 months)	Defensive freezing	13- and 24-months old rats displayed more freezing than 4-months old rats; gonadectomy in 4-months old rats, but not in 13- and 24-months old rats, increased freezing, which was reversed by chronic testosterone supplementation; a single injection of 3α-diol, but not testosterone, had anxiolytic effect regardless of the age
(41)	Rats (Lewis) Males	Middle-age (from 12 months)	In pre-pubertally gonadectomized rats: single injection of testosterone (1 mg/kg) single injection of estradiol (100 μg/kg) 2-week estradiol treatment (10μg/kg)	Middle-age (from 12 months)	Elevated plus maze	None of the treatment affected anxiety-related behavior in gonadectomized male rats
(23)	Mice (C57/B6) Males	Old-age (24 months)	Testosterone, 3α-diol or estradiol injected in aged intact mice (1 mg/kg)	Old-age (24 months)	Open field, light-dark box, elevated plus maze, zero maze, mirror chamber, Vogel conflict test	In gonadally intact aged testosterone and 3α-diol had anxiolytic effects in all of the conducted tests, while estradiol had anxiolytic effects in open field, light-dark box and mirror chamber only
(43)	Rats (Fisher) Females and males	Old-age (20 months)	Exposure to chronic restraint stress for 21 days	Old-age (21.5 months)	Open field, novel object exposure	Gender differences: males were generally more anxious than females stress increased male and decreased female anxiety-related behaviors, and had no effect on testosterone concentration

## Prenatal and neonatal period

Early development is a critical period for brain formation and also for its modulation by sex hormones contributing to sexual dimorphism. Prenatal exposure to testosterone excess is associated with impaired neural development and mental functions (44, 45). Offspring of women with polycystic ovary syndrome is exposed to high concentration of androgens *in utero*. As shown in a rat model, maternal hypergonadism may result in increased risk for developing anxiety disorder in offspring, especially in female offspring (39). Inhibition of testosterone aromatization to estradiol in pregnant dams may induce increased defecation and freezing in the open field, as well as decreased number of hangings from and time spent on the open arms, pointing out an increased emotionality and anxiety in both, female and male offspring (28, 46). Accordingly, exposure to high concentration of testosterone during prenatal development leads to anxiogenesis in the adulthood, and females seems to be more sensitive to this effect of prenatal testosterone.

In addition to prenatal, also perinatal exposure to testosterone may predetermine anxiety-related behavior later during life. As found in adult rats and mice, testosterone injected on the day of birth or neonatal ovariectomy can masculinize anxiety-related behavior of females increasing the number of the marbles buried (27) and decreasing the time spent on the open arms (47). On contrary, adult male rats, castrated on the day of birth have a higher locomotor activity in the open arena, spent more time in the center zone, in the light chamber, in the open arms, and exhibit a higher number of novel object visits than their sham operated counterparts (40, 48). These results suggest that the absence of estrogens in females and testosterone in males during the perinatal period restrain the normal differentiation of gender-specific adult anxiety responses to the particular stimuli.

## Adolescence

Puberty is a restricted period in adolescence characterized by a rapid elevation of circulating sex hormones accompanied by reproductive maturation. Adolescence, as the age between childhood and adulthood, represents a sensitive period in neurodevelopment as well. Numerous evidence proves that the remodeling of the adolescent brain occurs under the influence of sex hormones (36, 37). A large body of animal experiments has shown that sex differences in several anxiety tests arise from the organizational effects of testosterone at peri-pubertal age. In rats, male gonadectomy, either at puberty onset or shortly thereafter, results in lower anxiety in the open area, the behavioral response typical for females (29). Similar effects of pre-pubertal castration were found in the elevated plus maze and light-dark box, where adult male rats that had been gonadectomized prior to puberty exhibit a higher frequency of head-dipping and more time spent in the open arms or in the light part of the light-dark box (33). Environment-related social anxiety in males is organized also by pubertal sex hormones, where the aromatization of testosterone seems to be critical (49). In addition, middle- (12-months old) as well as old-aged male rats (30-months old) that have been castrated before puberty onset or at late adolescent age, respectively, display lower or similar anxiety in comparison to intact aged-matched females in a test-specific manner (41, 42). Pre-pubertal gonadectomy has anxiogenic consequences in male mice in the elevated plus maze, but anxiolytic effect in female mice in the marble-burying test (26). On the other side, testosterone exposure and social experience during adolescence equally increase anxiety-like behavior, as shown in adult male hamsters (50). In females rats, single testosterone administration at the day of birth (organizational effect) as well as continual testosterone exposure from the puberty onset (activational effect) increases activity in marble-burying test of anxiety-like behavior, abolishing sex differences in adulthood (27). Furthermore, while in 1-month-old intact rats no sex differences were found in anxiety-like behavior, testing at 3 months of age revealed clear sex differences in reaction to the environmental novelty, females being more active and less anxious than males in the open field and in the elevated plus maze (28). Interestingly, in late adolescence, male mice spent more time on open arms than females. However, this sex difference in open-arm time is not present prior to puberty onset and does not persist into young adulthood (26).

## Adulthood

Most published experiments examine the role of testosterone in anxiety-like behavior and the underlying mechanisms in young adult animals. Unlike the anxiolytic effect of peri-pubertal castration, castration of adult males is frequently associated with increased anxiety-like behavior in a battery of behavioral tests, such as open field, elevated plus maze, and defensive-burying test (51–58). However, in some experiments in rats, no effect of adult gonadectomy was found (29, 33). Presumably, the different time interval between hormone deprivation and behavioral examination among the studies may result in these differences. The short-term effect of gonadectomy has been investigated in a large number of experiments, while the effects of the long-term hypogonadism are understudied.

The anxiogenic effect of adult castration might be reversed by hormone supplementation (52, 54, 59–63). The anxiolytic-like effect of testosterone has been revealed in gonadally intact healthy animals, as well (64–66). However, testosterone can be either reduced by 5α-reductase to the more potent androgen dihydrotestosterone, or aromatized by aromatase to estradiol converting the androgen to estrogen activity. In brain, dihydrotestosterone can be further metabolized to 5α-androstane-3α,17β-diol (3α-diol) and to 5α-androstane-3β,17β-diol (3β-diol), neuroactive steroids possessing neuromodulatory activity (67). One of the most cited study investigating the rapid effects of testosterone on anxiety-like behavior in mice has suggested that the anxiolytic effect of testosterone is mediated by its 5α-reduced metabolites (64). Likewise, it has been proved that administration of 3α-diol decreases anxiety-like behavior in male rats (52, 59, 60, 63) as well as in female rats (68), while inhibition of testosterone metabolism to 3α-diol increases anxiety in male rats (62). Similar to effect of hormone administration, sexual experience and the exposure of intact male rats or mice to female subjects may decrease anxiety-like behavior associated with increased concentration of testosterone in plasma and hippocampus, as well as increased hypothalamic testosterone and 3α-diol concentration (64, 69). On the other hand, estradiol (61) and another metabolite of testosterone, androsterone (70), may cause anxiolysis, as well. Fernández-Guasti and Martínez-Mota (54) have shown that repeated administration of testosterone, but not a single injection of testosterone, nor treatment with 3α-diol or androsterone produced anxiolysis in male gonadectomized rats (54). According to published data, the manifestation of the anxiolytic-like effect of testosterone or its metabolites is highly dependent on dose and duration of treatment (54, 58, 64, 65, 71).

Due to its complex metabolism, the effects of testosterone might be mediated through different mechanisms of action. Testosterone and dihydrotestosterone are ligands of the androgen receptor. The latter binds with a greater affinity to the receptor and activates gene transcription resulting in an increased androgen activity (72). The role of androgen signaling in the regulation of anxiety-related behavior was demonstrated by administration of the androgen receptor antagonist flutamide (52, 54, 66), but also using the animal model of testicular feminization mutation (73–76) and androgen receptor knockout mice (77). In addition, it has been shown that some selective androgen receptor modulators may exert neuroprotective effects (78) and may affect some behavioral and brain functions (79–83). However, experimental studies examining their effects on anxiety important for the deeper understanding of the association between testosterone and anxiety, but potentially also for therapeutic applications are lacking. Testosterone can exert its effects also via estrogen receptors following aromatization of testosterone to estradiol, as well as by conversion of dihydrotestosterone to 3α-diol and 3β-diol (23, 71). Furthermore, it was shown that testosterone may modulate γ-aminobutyric acid (GABA)-stimulated chloride influx (84). This might be due to the fact that 3α-diol and androsterone can bind to GABA_A_/benzodiazepine receptor. Unlike to GABA_C_ receptor, which seems not to be involved in the regulation of anxiety by testosterone (85), inhibition of GABA_A_ receptor by bicucculine or picrotoxin (64), as well as by flumazenil (70) diminishes the anxiolytic effect of testosterone metabolites. In females, inhibition of GABA_A_ and ERβ receptor diminishes the anxiolytic effect of testosterone, but not when aromatase is inhibited. Thus, the anxiolytic effect of testosterone might be mediated by its reduced metabolites via GABA_A_ with participation of ERβ (86). On the other side, using βER-knockout mice, it has been shown that the anxiolytic effect of testosterone metabolites mediated by the GABA_A_ receptor may be not as robust as via activation of ERβ receptor (70). In addition, it was shown that testosterone can regulate serotonin neurotransmission. Gonadectomy results in decreased expression of 5-HT_2A_ serotonergic receptors in hippocampus, testosterone replacement enhances 5-HT_2A_ expression accompanied by reduced anxiety (58). On the contrary, inhibition of 5-HT_1A_ receptor increases efficacy of testosterone treatment leading to enhanced anxiolytic-like effect (53). As shown by observation of inter-individual differences, anxious animals display higher expression of tryptophan hydroxylase—an enzyme involved in serotonin synthesis, and higher concentration of serotonin in amygdala than their non-anxious counterparts (35). Interestingly, while in gonadally intact rats, anxiety-like behavior positively correlates with the expression of tryptophan hydroxylase, this association is abolished by gonadectomy. As serotonin depletion diminishes anxiety level in anxious individuals, it also reverses the anxiogenic effect of gonadectomy (57).

## Aging

Aging is associated with a progressive decline of circulating testosterone resulting from decreased function of the hypothalamo-pituitary-gonadal axis. Unlike menopause in women, andropuase in men is a long-lasting process. The age-related hypogonadism in men is considered to cause some of the senescence symptoms, including anxiety (87). This has been proved in rats. While gonadectomy in young adulthood and, partially, at middle-age induces anxiety, this effect is less apparent in aged rats (22). On the other hand, in a genetic mouse model of aging, no association was found between low testosterone and anxiety (88). It should be considered that andropause is via negative feedback associated with increased concentration of luteinizing hormone. In adult female rats, agonist of gonadotropin-releasing hormone triptorelin, which inhibits luteinizing hormone release, alone as well as in combination with estradiol treatment reduces gonadectomy-induced anxiety (89). Further experiments are needed to examine the impact of luteinizing hormone modulation on anxiety in aging males.

In our laboratory, sex differences in anxiety-like behavior of middle-aged (12-months old) rats have been shown. Aging females were less anxious than aging males in a battery of behavioral tests (41). Similarly, male rats at older age (21-months old) have proved to be generally more anxious than females. In addition, stress in males increased, while in females it decreased anxiety-like behavior (43). However, we have shown that at very old age (30-months old) female and male rats do not differ any more in anxiety-like behavior in elevated plus maze and light-dark box, or in open-field ambulation, except rearing behavior (42). These results suggest that the causal role of endogenous testosterone in anxiety-related behavior at is doubtful at least in aging animals. However, it should be considered that the anxiety-like behavior in these rats might be biased by age-related decline in locomotor activity (90). Presumably, the sex differences, observed in young and middle-aged animals, result from the organizational effect of testosterone occurring at early development and puberty. These differences, however, do not persist into the old age, which can be caused by age-related cognitive and affective decline.

There is some evidence suggesting the anti-anxiety properties of exogenous testosterone as well as its metabolites, 3α-diol and estradiol, in old gonadally intact rats (23, 91) or in rats gonadectomized at old age (22). On contrary, we have demonstrated that neither single administration of testosterone or estradiol, nor short-term treatment with estradiol affect anxiety-like behavior in middle-aged rats, when they suffer from long-term hypogonadism initiated before puberty onset (41). Therefore, it seems to be possible that the length of testosterone deficiency as well as the age of animals might determine the activational effect of supplemented hormones on anxiety-like behavior. Furthermore, the age, dose and duration of hormone replacement therapy as well as their interactions were shown to influence the action of testosterone leading to either anxiolysis or anxiogenesis (91).

## Conclusion

Sex differences in anxiety-like behavior origin from organizational effect and might be modified by activational effect of sex hormones. There are critical periods across life that are crucial in organization of neuronal circuits and pre-programming the activational effect of testosterone on anxiety-like behavior. According to the current experimental studies, testosterone exposure during brain development predetermines higher anxiety in males. Furthermore, the absence of testosterone during the activation of neuronal circuits involved in anxiety results in anxiogenesis, as well. Although it seems that testosterone might have anxiolytic effect in adult males following short-term androgen deficiency, the impact of testosterone on anxiety-related behavior following long-term hypogonadal condition is understudied, mainly in aged animals, and needs further examinations. Based on the experimental results, age is a crucial factor that modulates the effect of both, endogenous and exogenous sex hormones. Thus, studying the expression of the various androgen receptors in different brain regions during development will be important to understand the contradictory findings from different experiments. Testosterone exposure or even deficiency in critical periods of development may have long-lasting consequences persisting into middle- or old age. This should be considered when interpreting experimental results and potentially hormonal treatments in human patients.

## Author contributions

ED drafted the manuscript, prepared the table and the figure. JH corrected the draft. DO corrected the draft. PC designed the review and corrected the draft. All authors approved the final version of the manuscript.

### Conflict of interest statement

The authors declare that the research was conducted in the absence of any commercial or financial relationships that could be construed as a potential conflict of interest.
